# Long-term impact of emergency laparotomy on health-related quality of life

**DOI:** 10.1007/s00068-024-02745-y

**Published:** 2025-01-24

**Authors:** Lív í Soylu, Dunja Kokotovic, Madeline Kvist, Jannick Brander Hansen, Jakob Burcharth

**Affiliations:** 1 Emergency Surgery Research Group Copenhagen (EMERGE), Department og Hepatic and Gastrointestinal Diseases, Copenhagen University Hospital– Herlev and Gentofte, Herlev, 2730 Denmark; 2 Department of Clinical Medicine, University of Copenhagen, Copenhagen, Denmark

**Keywords:** Emergency surgery, Laparotomy, Health-related quality of life, Readmission, Surgical outcomes

## Abstract

**Purpose:**

Emergency laparotomy can result in a range of physical and neuropsychiatric postoperative complaints, potentially impacting quality of life. This study aimed to assess the effect of emergency laparotomy on health-related quality of life (HRQoL) and how HRQoL influences the risk of readmission.

**Method:**

HRQoL was assessed in patients undergoing emergency laparotomy during a 1-year period. Patients who completed the baseline HRQoL evaluation underwent a reassessment on postoperative day (POD) 30, 90, and 180. HRQoL was measured with the EQ5D index, and patients were categorized in ‘high’ and ‘low’ HRQoL. A decrease from high baseline HRQoL to low HRQoL by POD 30 was classified as ‘acquired low HRQoL’.

**Results:**

All 215 patients who completed the baseline HRQoL evaluation were followed. On average, patients reported a lower mean (M) HRQoL from baseline (M = 0.876, standard deviation (SD) = 0.171) to POD 30 (M = 0.735, SD = 0.260). On POD 90, HRQoL had somewhat improved (M = 0.763, SD = 0.298), and by POD 180 HRQoL had returned to normal (M = 0.853, SD = 0.235). From the full-record population (*n* = 73), 20.5% acquired low HRQoL of whom 33% had not recovered by POD180. For patients with acquired low HRQoL, the risk of 180-day readmission was increased, and days alive and out of hospital within 180 days was reduced.

**Conclusion:**

For most patients, HRQoL has returned to normal within 180 days after emergency laparotomy. However, patients who acquired low HRQoL after the procedure had an increased risk of long-term readmission.

**Supplementary Information:**

The online version contains supplementary material available at 10.1007/s00068-024-02745-y.

## Introduction

Emergency laparotomy is a common procedure performed in patients suffering from bowel obstruction, perforation, trauma, bleeding, and mesenteric ischemia [1]. At the time of surgery, patients are critically ill, most often suffering from severe volume depletion and/or sepsis, leading to organ dysfunction [2]. Both the critical illness and the inflicted surgical injury are causing an inflammatory response, leading to impaired immune function and muscle wasting, resulting in compromised recovery [3]. In addition, patients undergoing emergency laparotomy are often elderly and frail, and a substantial fraction are suffering from reduced physical capability before surgery, a known predictor of delayed recovery [4–6]. However, there is limited information on the long-term general well-being of patients following emergency laparotomy.

Health-related quality of life (HRQoL) is a multi-dimensional concept used to measure how patients’ subjective perception of health affects quality of life. Several patient-reported outcome measure (PROM) instruments examining quality-of-life have been developed and validated in the general population and commonly explore physical function, mental well-being, and independence [7]. In the surgical field, postoperative HRQoL is often used to measure the burden of late sequelae and the quality of recovery. Recovery is multifactorial and depends on preoperative physical performance, pre-existing comorbidities, the inflicted surgical stress, as well as post-surgical complications [6, 8–10]. The majority of patients undergoing emergency laparotomy experience a deterioration of HRQoL after surgery [8], however, it has not been thoroughly examined how patient-reported HRQoL after emergency laparotomy affects recovery and surgical outcomes [8].

This study examined how undergoing emergency laparotomy affects patient-reported HRQoL and its impact on long-term readmission.

## Methods

### Study population

This prospective single-center study was set at Copenhagen University Hospital, Herlev, Denmark. In Denmark, public hospitals and healthcare services are financed by general taxes, providing free and equal access to healthcare for all Danish citizens. The healthcare system is divided into five regions, each responsible for operating the hospitals within the region. Copenhagen University Hospital, Herlev, is located in the Capital Region, serving 1,900,000 patients [11, 12]. The hospital serves a population of 465,000 patients and performs approximately 400 emergency laparotomies annually in approximately 300 patients (including second look operations, reoperations, abdominal traumas, and recurring operations). The surgical department has implemented a pre- and intraoperative bundle-of-care approach for patients presenting with high-risk surgical conditions [13]. Furthermore, recovery takes place in a dedicated emergency surgery ward, with standardized postoperative care and a focus on enhanced recovery [14].

From 1 August 2021 to 31 July 2022 baseline HRQoL was assessed in 215 adult patients (≥ 18 years of age) undergoing primary or secondary emergency laparotomy due to bowel obstruction, bowel perforation, ischemia, anastomotic leakage, postoperative bleeding, or fascial dehiscence at Copenhagen University Hospital, Herlev [15]. Patients who underwent emergency laparotomy due to trauma were excluded. All 215 patients who participated in the baseline HRQoL assessment were eligible for inclusion in this study.

The study was approved by the Danish Data Protection Agency and the Capital Region of Copenhagen (P-2021-431, P-2020-1166). The study did not qualify for ethics approval by Danish law as no intervention was carried out.

### Health-related quality of life assessment

The HRQoL was assessed with the EQ5D index, a widely used instrument measuring patient-perceived HRQoL, developed by the EuroQol group [16]. The EQ5D index comprises a short descriptive system questionnaire (EQ5D5L) and a visual analog score (EQ-VAS). The EQ5D5L illustrative system includes five dimensions: mobility, self-care, usual activities, pain/discomfort, and anxiety/depression. Each domain is scored on a five-point scale: 1, no problem; 2, slight problem; 3, moderate problem; 4, severe problem; 5, unable to do/extreme problem. The 5L responses are converted to a value (i.e. utility score) using a preference-based scoring system derived from the general Danish population [17, 18]. The EQ-VAS scale measures patient-perceived overall health from 0 to 100, with 0 being the worst imaginable health and 100 the best. The EQ-VAS was not assessed in this study.

For this study, all patients who had completed a baseline HRQoL assessment during inclusion (*n* = 215) received a phone call on POD 30, 90, and 180, comprising a re-evaluation of HRQoL according to their current self-perceived health. When patients were out of reach, a second contact attempt was made the following days. If the second attempt failed, patients were not called again until the time for the next follow-up.

### Readmission and days alive and out of hospital (DAOH)

Information on patient characteristics, details regarding the index admission, date and cause of unplanned readmissions, and date of death were retrieved from the electronic patient records. In 2017, the Capital Region and the neighboring region (the Region of Zealand), joined electronic health records, granting health professionals access to patient records from all hospitals in the area [19]. All unplanned readmissions in the period POD 30 (time of first evaluation) to POD 180 (time of last evaluation) were counted and registered with a primary cause and length of stay (LOS) to examine the effect of HRQoL on POD 30 on readmission.

Days alive and out of the hospital at 90 (DAOH_90_) and 180 days (DAOH_180_) were calculated. The day of surgery was day 0, and all subsequent days in-hospital or deaths were subtracted from the study period of 90 or 180 days. Both total and part-days in the hospital were subtracted; thus, the maximum possible values for DAOH_90_ and DAOH_180_ were 89 and 179, respectively. This method is described in detail [20–22].

### Analyses

Based on the utility scores from the EQ5D, patients were categorized into high HRQoL (utility scores 1.0–0.80), and low HRQoL (utility scores < 0.8) at baseline and during the three follow-up times. Patients with a baseline utility score equal to or above 0.80 (i.e. high baseline HRQoL), who at POD 30 reported a utility score below 0.8 (i.e. low HRQoL) were considered to have an ‘acquired low HRQoL’.

Categorical data are presented as cases and percentages. Differences (for both binary and polytomous variables) between patients with low and high HRQoL were analyzed using Pearson’s chi-square test and Fisher’s exact t-test. The distribution of continuous data (variables age and length of stay) was assessed by visual inspection of histograms, and the median and interquartile (IQR) ranges were calculated. The Mann-Whitney U test was used for comparison. The reoperation and readmission rate was defined as the number of patients with a least one reoperation or readmission in the given period.

A Kaplan Meier failure plot was performed for unplanned readmission from the first time of follow-up (POD 30) to the last time of follow-up (POD 180), according to HRQoL. The time of failure was first readmission (outcome) or censoring at death or 180 days after discharge, whichever came first.

A cause-specific Cox proportional hazard regression was performed to identify independent risk factors for readmission during the investigational period. Days from operation were used as the underlying time scale, and patients were followed from POD 30 to POD 180. Based on current literature, relevant clinical variables were included in the analysis: HRQoL on postoperative day 30 (high/low), sex (male/female), WHO performance status (0–1/≥2), living alone (yes/no), any postoperative complication with a Clavien-Dindo Classification score ≥ 2 (yes/no), and discharge with in-home assistance or to a rehabilitation facility (yes/no).

All analyses were performed using SAS software, version 9.4. SAS Institute Inc., Cary, NC, USA.

## Results

### Populations

**Fig. 1:**
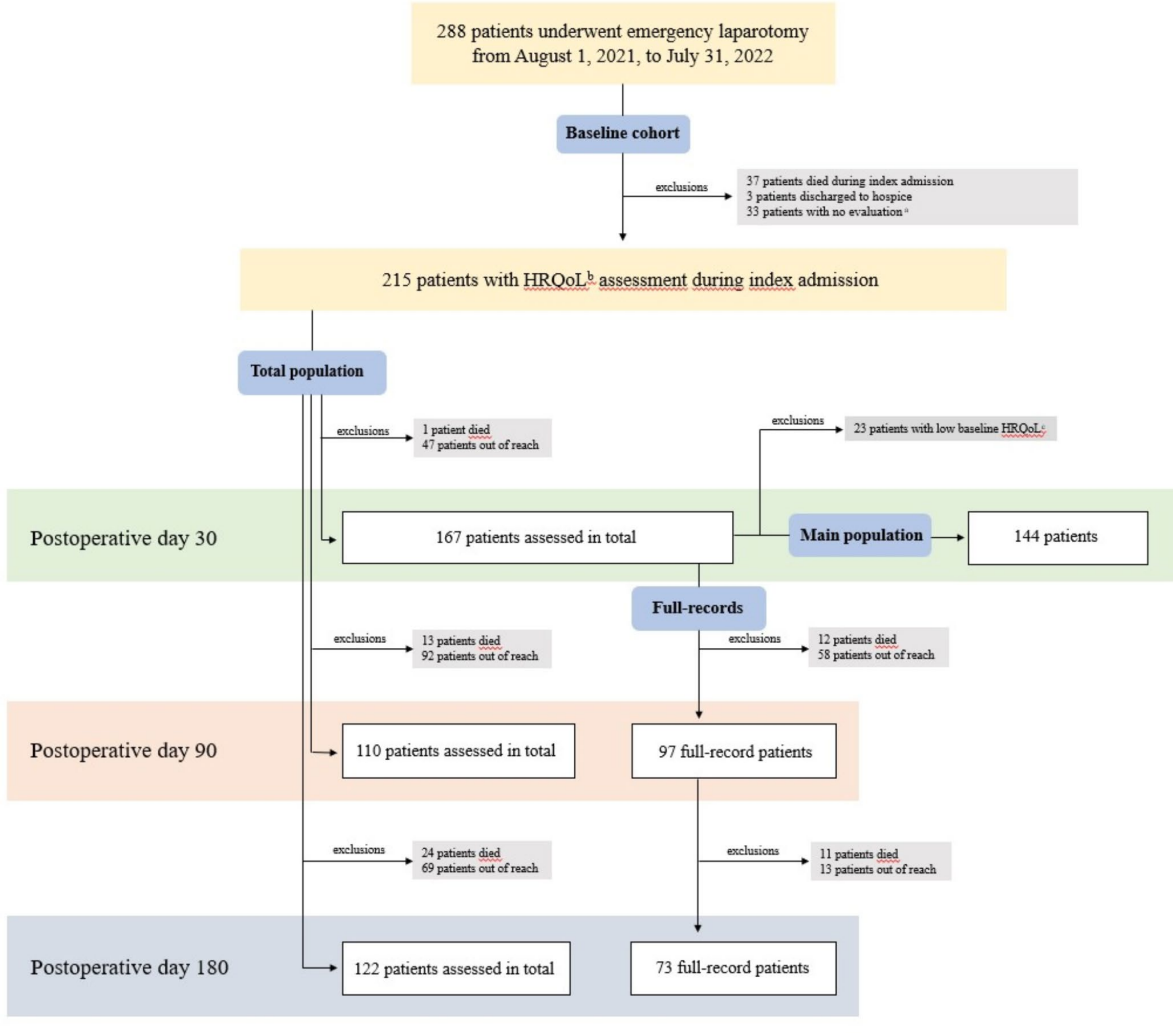
An overview of the study population. ^a^Due to patient was unwilling or unable to participate, or discharged prior to evaluation. ^b^Health-related Quality of Life. ^c^Patients with low self-reported health-related quality of life at baseline (index admission) and at postoperative day 30, also see supplemental Fig. 1

From August 1, 2021, to July 31, 2022, baseline HRQoL was evaluated in 215 patients, forming the *baseline cohort* (Fig. 1). From the baseline cohort, HRQoL was assessed in 167 patients on POD 30, 110 patients on POD 90, and 122 patients on POD 180, forming the *total populations* at each follow-up time. On POD 180, HRQoL was assessed in 73 patients at all three follow-up times (POD 30, 90, and 180), forming the *full-record population* (Fig. 1). From the *total 30-day population*, the *main population* comprising 144 patients was formed, excluding patients with low baseline HRQoL combined with low HRQoL on POD30 (*n* = 23). This way, we were able to examine patients with a high HRQoL after emergency laparotomy opposed to patients who acquired low HRQoL after emergency laparotomy (Fig. 1). In the Supplemental Fig. 1, categorization according to high and low HRQoL during follow-up is graphically presented.

Baseline characteristics are demonstrated for the total 30-day population (Table 1) and for the main population according to HRQoL on POD30 (Table 2). Patients who acquired low HRQoL on POD 30 were more often females, were more likely to live alone, had a higher ASA score and a lower performance score on average, more often suffered postoperative complications, were more likely to be discharged with in-home assistance or to a rehabilitation facility and had longer LOS of index admission.

**Table 1:** Baseline characteristics in the total 30-day population (*n* = 167)

	Total 30-day population ^a^
Total, no (%)	167
Female sex, no (%)	81 (48.5)
Age, no (%)	
<70	84 (50.3)
70–80	55 (32.9)
≥80	28 (16.8)
Median (IQR)	69 (56–77)
Living alone, no (%)	64 (38.6)
Active smoking, no (%)	38 (23.0)
Excessive alcohol consumption, no (%) ^b^	25 (15.4)
ASA score, no (%)	
1–2	104 (62.3)
3–5	63 (37.7)
WHO Performance Score, no (%)	
0–1	133 (79.6)
≥2	34 (20.4)
Stoma placement, no (%)	35 (21.0)
Complications	
Any postoperative complication, no (%)	96 (57.5)
CD score ≥ 2, no (%) ^c^	75 (44.9)
ICU admittance, no (%) ^d^	25 (15.0)
LOS, median (IQR) ^e^	8 (5–12)
Discharge with in-home assistance or to rehabilitation facility, no (%)	55 (32.9)
30-day reoperation rate, no (%) ^f^	39 (23.4)
30-day readmission rate, no (%) ^g^	46 (27.5)

^a^For a graphic presentation of the population, see Fig. 1 and Supplemental Fig. 2a

^b^More than 21 or 11 units of alcohol for men and women, respectively

^c^Clavien-Dindo Classification of surgical complications

^d^Intensive care unit

^e^Length of stay from operation to discharge

^f^Number of patients with at least one reoperation within 30 days from surgery

^g^Number of patients with at least one emergency readmission within 30 days from surgery

**Table 2:** Baseline characteristics in the main population (*n* = 144) according to HRQoL on postoperative day 30

	Main population^a^	High HRQoL^b^	Acquired low HRQoL^b^	*p*-value
Total, no (%)	144	96	48	
Female sex, no (%)	70 (48.6)	41 (42.7)	29 (60.4)	0.045
Age, no (%)				0.707
<70	73 (50.7)	51 (53.1)	22 (45.8)	
70–80	47 (32.6)	30 (31.3)	17 (35.4)	
≥80	24 (16.7)	15 (15.6)	9 (18.7)	
Median (IQR)	69 (56–77)	68 (56–76)	71 (56–78)	0.717
Living alone, no (%)	50 (35.0)	27 (28.4)	23 (47.9)	0.021
Active smoking, no (%)	30 (21.1)	16 (16.8)	14 (29.8)	0.075
Excessive alcohol consumption, no (%) ^c^	21 (15.1)	13 (13.9)	8 (17.4)	0.597
ASA score				0.0027
1–2	96 (66.7)	72 (75.0)	24 (50.0)	
3–5	48 (33.3)	24 (25.0)	24 (50.0)	
WHO Performance Score				0.0005
0–1	123 (85.4)	89 (92.7)	34 (70.8)	
≥2	21 (14.6)	7 (7.3)	14 (29.2)	
Stoma placement, no (%)	30 (20.8)	16 (16.7)	14 (29.2)	0.082
Complications				
Any postoperative complication, no (%)	79 (54.9)	45 (46.9)	34 (70.8)	0.007
CD score ≥ 2 ^d^	62 (40.1)	33 (34.4)	29 (60.4)	0.003
ICU admittance, no (%) ^e^	20 (13.9)	12 (12.5)	8 (16.7)	0.496
Discharge with in-home assistance or to rehabilitation facility, no (%)	42 (29.2)	20 (20.8)	22 (45.8)	0.002
LOS, median (IQR) ^f^	7 (5–11)	6 (4–9)	9 (6–15)	0.0001
30-day reoperation rate, no (%) ^g^	34 (23.6)	20 (20.8)	14 (29.2)	0.267
30-day readmission rate, no (%) ^h^	39 (27.1)	25 (26.0)	14 (29.2)	0.691

^a^For a graphic presentation of the population, see Figure 1 and Supplemental Figure 2b

^b^High HRQoL (scores 1.0-0.80). Low HRQoL (scores < 0.80)

^c^More than 21 or 11 units of alcohol for men and women, respectively

^d^Clavien-Dindo Classification of surgical complications

^e^Intensive care unit

^f^Length of stay of index admission

^g^Number of patients with at least one reoperation within 30 days from surgery

^h^Number of patients with at least one emergency readmission within 30 days from surgery

### HRQoL following emergency laparotomy

**Fig. 2:**
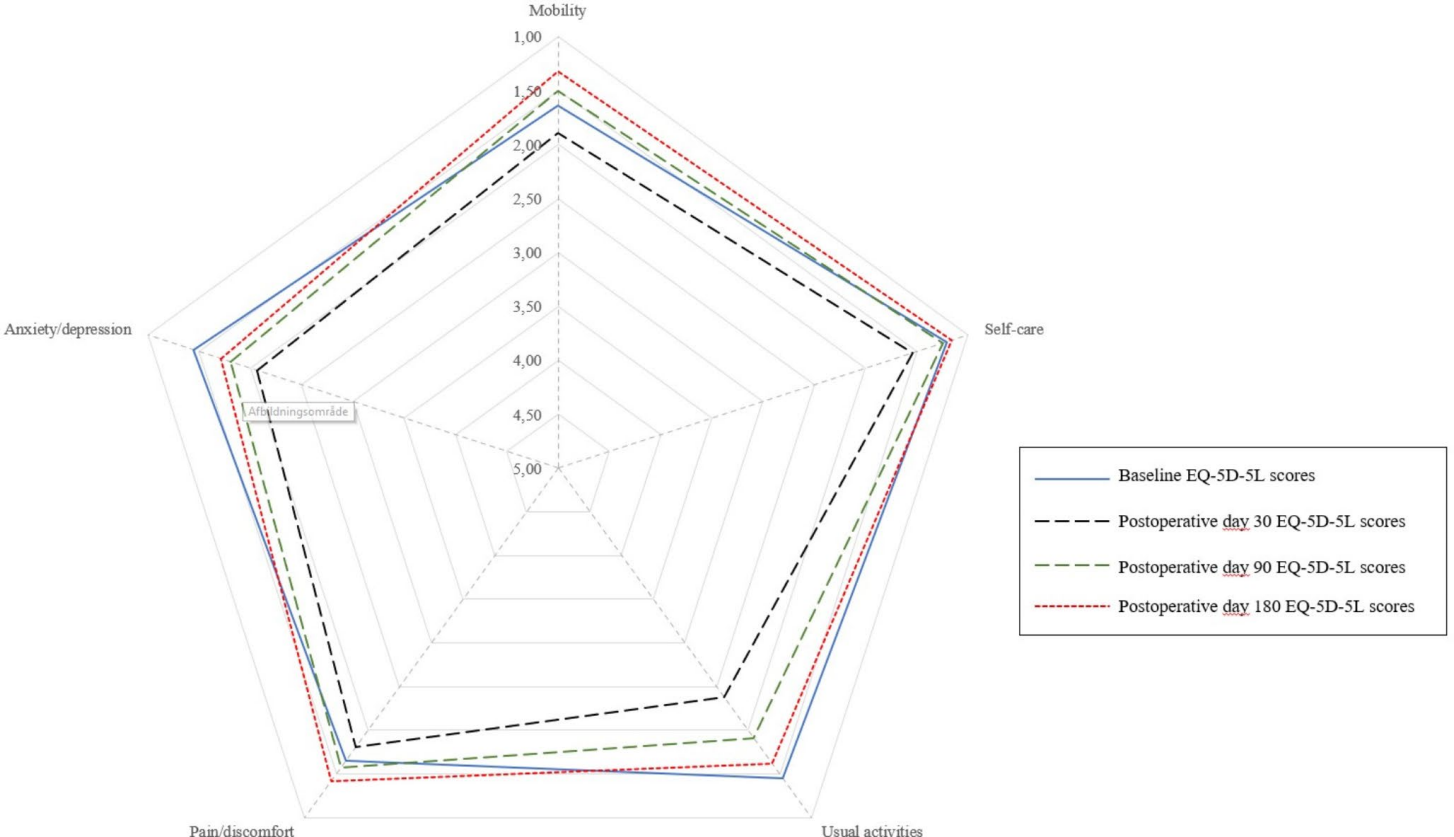
Graphic presentation of EQ5D domain responses at baseline, postoperative day 30, 90 and 180. The EQ5D5L domains are scored on a 5-point scale: 1, no problem; 2, slight problem; 3, moderate problem; 4, severe problem; and 5, unable to do

For the total populations, the mean baseline HRQoL was 0.876 (SD = 0.171) and declined to 0.735 (SD = 0.260) on POD 30. On POD 90, the mean HRQoL improved to 0.763 (SD = 0.298), and on POD 180, the mean furtherly improved to 0.853 (SD = 0.235). Patients reported poorer functioning in all five domains (i.e., mobility, self-care, usual activities, pain/discomfort, depression/anxiety) on POD 30 compared to baseline reports. On POD 90, patients reported an improved level of functioning in mobility, self-care, and pain/discomfort, yet still reported problems performing usual activities and depression/anxiety (Fig. 2). By POD180, patients reported an overall similar level of functioning in all five domains compared to the baseline HRQoL (Fig. 2).

In the full-record population (*n* = 73), 15 (20.5%) patients acquired low HRQoL on POD 30. On POD 90, six (40%) out of 15 patients had recovered to a high HRQoL, and on POD 180, the number accumulated to 10 (66%) patients, meaning that 33% of patients who acquired low HRQoL had not recovered by POD 180, and 7% from the total full-record population.

### Readmission and DAOH

In the main population, 28 (14.3%) patients were emergently readmitted from POD 30 to POD 90, accumulating to 40 (20.4%) patients by POD 180.

**Fig. 3:**
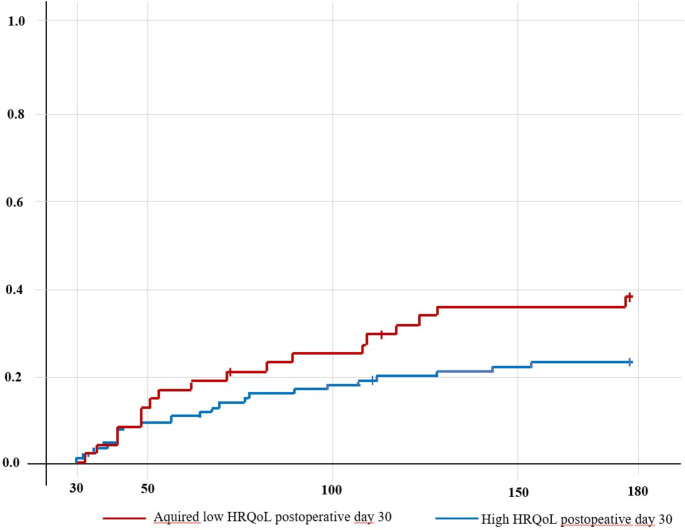
Readmission from postoperative day (POD) 30 to POD 180 for the main population (*n* = 144) according to health-related quality of life on POD 30

For patients who acquired low HRQoL on POD 30, there was a tendency towards a higher subsequent emergency readmission rate; however, it was not significant (*p* = 0.066). Nevertheless, in a failure analysis examining readmission according to HRQoL on POD 30, patients who acquired low HRQoL seemed to have an increased risk of readmission from approximately POD50 on onwards (Fig. 3). Acquiring low HRQoL was also associated with fewer DAOH within both 90 and 180 days (*p* = 0.0004 and *p* = 0.0001, respectively) (Table 3).

**Table 3:** Readmission risk in the main population for patients with acquired low health-related quality of life (HRQoL) on POD30

	Population	High 30-day HRQoL	Acquired low 30-day HRQoL	*P*-value
Readmission, no (%)	144	96 (66.7)	48 (33.3)	
30–90 days ^a^		16 (16.7)	12 (25.0)	0.230
30–180 days ^a^		18 (37.5)	22 (22.9)	0.066
Days alive and out of hospital, median (IQR)	144			
90 days		83 (78–85)	79 (64–82)	0.0004
180 days		172 (166–175)	167 (151–172)	0.0001

^a^Readmission from postoperative day (POD) 30 to postoperative day 90 or 180

^b^Health related Quality of Life according to high HRQoL (scores 1.0-0.80) and low HRQoL (scores < 0.80)

We examined if patients with a low baseline HRQoL (*n* = 39) were more likely to be readmitted compared to patients acquiring low HRQoL on POD 30 (*n* = 48) and found that there was no difference in the number of readmissions between the two populations (Supplemental Fig. 2c portrays the population examined).

In a Cox proportional hazard regression model, discharge with in-home assistance or rehabilitation was an independent risk factor for emergency readmission from POD30 to POD180 (HR = 2.70, 95% CI = 1.27–5.75, *p* = 0.0098). Independent risk factors are presented in Supplemental Table 1.

### Mortality

Out of the total of 24 patients who died during follow-up, 23 died *after* the time of the first follow-up (Fig. 1). For patients who died during follow-up, there was no notable difference in the number of patients with high and low HRQoL on POD 30 (5 [5.2%] and 5 [7.0%], respectively). However, more patients who died had missed a follow-up before death, and only ten out of the 23 patients alive at POD 30 evaluated HRQoL on POD 30.

## Discussion

In this prospective study examining the impact of HRQoL after emergency laparotomy on long-term readmission, we found that the majority of patients experienced a temporary decline in HRQoL; however, they recovered within 180 days. For the 20% of patients who *acquired* low HRQoL after emergency laparotomy, both temporary and long-lasting, the risk of 180-day readmission was increased.

The results from a recent review support that HRQoL for the vast majority of patients undergoing emergency laparotomy, returns to normal within three months of surgery [8]. In our study, HRQoL had improved at three months, yet patients still reported problems with depression/anxiety and problems performing usual activities. In a survey of overall recovery after major abdominal surgery, examining everyday living, physical performance, and mental health, up to 50% of patients had not recovered to pre-existing level of functioning within six months [6]. Nevertheless, most patients had recovered the ability to care for themselves and manage basic affairs within the first three months but were still experiencing difficulties in performing more strenuous activities and tasks requiring a higher level of cognition, in line with our findings [6].

In our study, 7% of patients reported a long-lasting deterioration of HRQoL after emergency laparotomy, which adds to the list of late sequelae described in current literature, such as chronic pain (one out of five), reduced functional ability (one out of three), change in employment (one out of five patients in the working force), and recurrent readmissions (one out of two within 180 days) [23–26].

Patients in our study who acquired low HRQoL after emergency laparotomy were vulnerable before surgery (i.e. high ASA-score, low performance) and were often subjected to serious postoperative complications, leading to prolonged LOS and the need for post-discharge rehabilitation. Although not statistically significant, our results suggest that acquiring low HRQoL after emergency laparotomy puts the patient at higher risk for long-term readmission. In a recent study, preoperative low HRQoL was an independent risk factor for readmission [15], and in accordance, we found that the risk of readmission was similar for patients with low baseline HRQoL and patients who acquired low HRQoL postoperatively. Although we do not know the specific mechanisms causing the association, we speculate that the patient’s perception of health is a reflection of the overall burden of disease.

For this study, the generic EQ5D instrument was used to examine quality of life. The EQ5D is the most commonly used instrument examining quality of life following emergency laparotomy, presumably due to its brevity and simplicity [27]. Other frequently used quality-of-life instruments are the generic SF-36 and the shorter SF-12, although more detailed, they are also more complex, a disadvantage in the emergency setting, where patients often have difficulties engaging with complex instruments due to impaired cognition [27, 28]. The reported long-term challenges following emergency laparotomy (i.e. pain, physical and mental dysfunction) are explored in the EQ5D [23, 24]. However, some patients undergoing emergency laparotomy are reporting long-term gastrointestinal issues, not covered by the EQ5D. To explore how gastrointestinal issues affects quality of life a symptom-specific quality-of-life instrument exploring bowel movements (e.g., the gastrointestinal quality of life index, GIQLI) could have supplemented the EQ5D, nevertheless, the GIQLI is a 36-item questionnaire, and therefore not well suited in the emergency surgery setting [29].

There were some limitations to this study. Our sample size for patients with full record information was limited (*n* = 73), which may lead to type II error. However, for the main analyses, the sample size was larger (*n* = 144), diminishing this risk. Furthermore, we were not able to get hold of all patients telephonically, which can lead to selection bias. However, several attempts of contact were made for each patient, minimizing the loss to follow-up. We did not have information on HRQoL during the earliest postoperative period (e.g., on POD7 or POD14) and, therefore, could not examine how emergency laparotomy affects HRQoL during the early postoperative days. However, measuring patient-reported HRQoL for patients still in the hospital, struggling with early postoperative recovery would not be a good measurement for recovery, as all patients undergoing emergency laparotomy, in one way or another, experience limitations during the immediate postoperative period.

The prospective design and detailed information on patient characteristics strengthen the study. Furthermore, we had the resources to contact patients via telephone, rather than the less resource-demanding electronic or paper questionnaire, increasing the response rate [30]. Information on readmission and death was available for all patients, allowing for time-dependent analyses and excluding patients who were unreachable due to death at the time of follow-up.

Our research supports that HRQoL is an important factor in recovery after emergency laparotomy. How patients experience recovery is very much influenced by functional ability, and what matters the most for a satisfactory quality of life, is returning to own home and remaining independent [24, 31, 32]. Patients suffering from comorbidities and pre-existing limited functioning have the highest risk of acquiring low HRQoL after surgery and are more often faced with a poor quality of recovery, inducing a vulnerability for a health deterioration resulting in repeated readmissions [3, 6, 24, 26]. Addressing HRQoL in discharge planning could help us identify the patients with the highest risk of permanently reduced HRQoL and long-term ill health and offer targeted screening for this selected group in form of telephonic follow-up on post discharge day 3, 7, 14 and 30, addressing pain management, bowel function, mental and physical wellbeing, with the option of prescribing in-home physical therapy, day-to-day consults in the ambulatory setting, or facilitating contact with a primary physician, based on each patients experiencing. This way, we could implement early preventive measures to minimize the risk of a long-term impaired HRQoL and dependency, increase patient well-being, and lengthen time in own home while allocating hospital resources to the patients that need them the most.

In conclusion, undergoing emergency laparotomy introduces a temporary decline in HRQoL, which for the vast majority returned to normal within 180 days. For patients acquiring low HRQoL postoperatively, the risk of 180-day emergency readmission increased slightly. By taking HRQoL into account in discharge planning, we would be able to identify patients who could benefit from targeted rehabilitation to improve recovery, general well-being, and quality of life.

## Electronic supplementary material

Below is the link to the electronic supplementary material.

Supplementary Material 1

Supplementary Material 2

Supplementary Material 3

## Data Availability

No datasets were generated or analysed during the current study.
