# Supplementary material for: Long-term impact of emergency laparotomy on health-related quality of life
Source: Eur J Trauma Emerg Surg. 2025 Jan 24;51(1):40. doi: 10.1007/s00068-024-02745-y (PMC11761775; doi:10.1007/s00068-024-02745-y)
Supplement: Supplementary file 1 — Supplementary Material 1 [file 68_2024_2745_MOESM1_ESM.docx]

| **Supplemental table 1. Independent risk factors for emergency readmission from 30 to 180 days** | | |  |
| --- | --- | --- | --- |
|  | 30-180-day follow-up | |  |
|  | HR ^a^ (95% CI ) ^b^ | p-value |  |
| Health-related Quality of Life ^c^ *(ref = High HRQoL POD30)* |  |  |  |
| Low HRQoL POD30 | 1.80 (0.89-3.63) | 0.102 |  |
| Male *(ref = female)* | 1.97 (0.99-3.95) | 0.054 |  |
| Who Performance Status ≥2 *(ref = PS 0-1)* | 1.20 (0.53-2.71) | 0.665 |  |
| Living alone *(ref = cohabiting)* | 0.87 (0.44-1.70) | 0.678 |  |
| Complication CD score ≥2 during index admission ^d^ *(ref = no)* | 0.61 (0.29-1.28) | 0.192 |  |
| Discharge to other than home ^e^ *(ref = discharge to own home without assistance)* | 2.70 (1.27-5.75) | 0.0098 |  |
| *^a^ Hazard ratio*  *^b^ 95% Confidence interval*  *^c^ Health-related Quality of Life (HRQoL) on postoperative day 30 and categorized in high HRQoL (utility scores 1.0-0.80),*  *and low HRQoL (utility scores < 0.80).*  *^d^ Clavien-Dindo Classification of surgical complications*  *^e^ Discharge to own home with in-home assistance or discharge to a rehabilitation facility* | | | |
