# Supplementary figures and images for: Long-term impact of emergency laparotomy on health-related quality of life
Source: Eur J Trauma Emerg Surg. 2025 Jan 24;51(1):40. doi: 10.1007/s00068-024-02745-y (PMC11761775; doi:10.1007/s00068-024-02745-y)

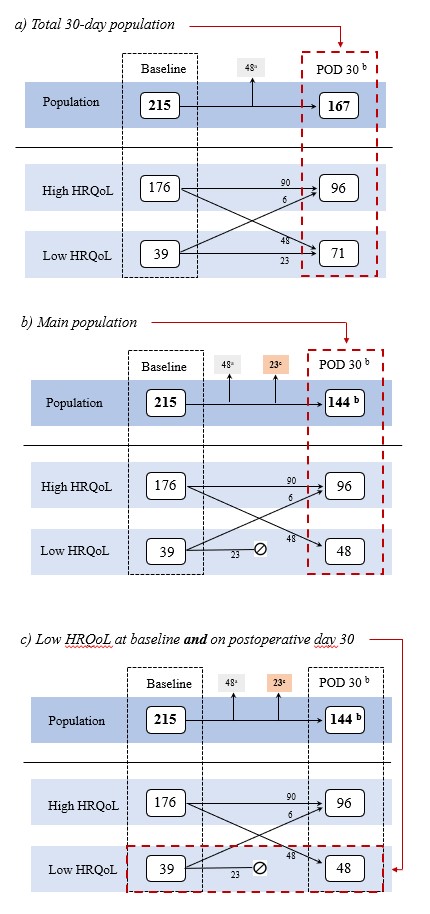

Supplement: Supplementary file 2 — Supplementary Material 2 [file 68_2024_2745_MOESM2_ESM.jpg]

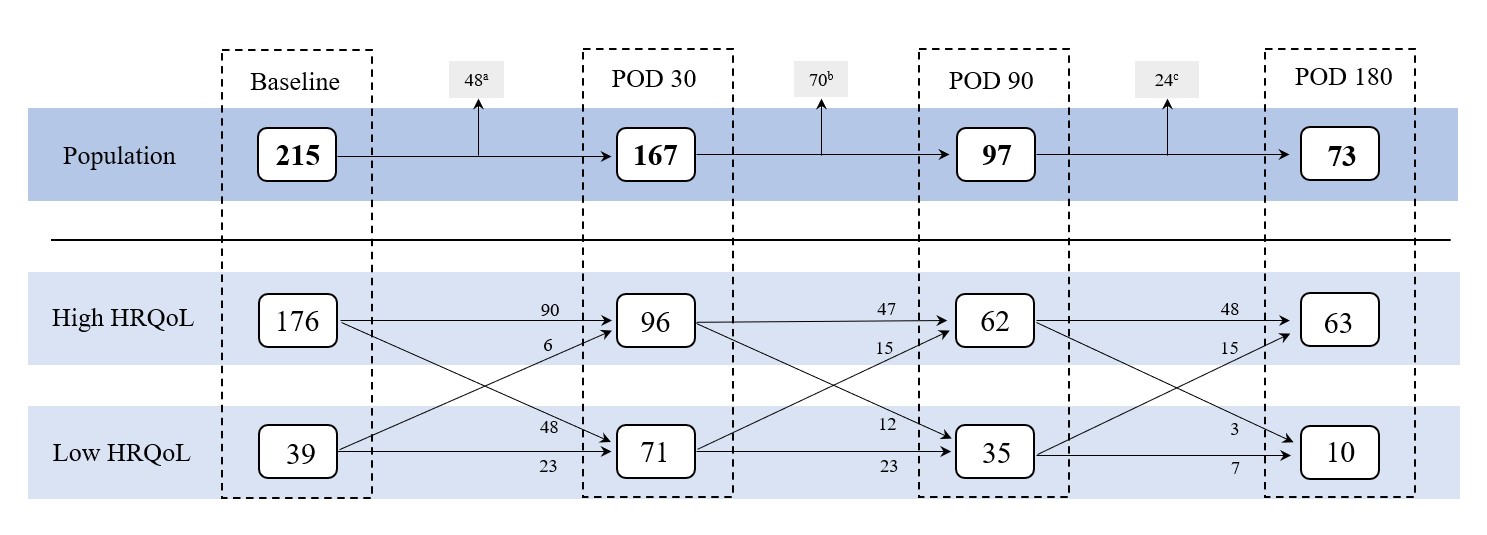

Supplement: Supplementary file 3 — Supplementary Material 3 [file 68_2024_2745_MOESM3_ESM.jpg]
